# Healthcare utilization of lung cancer patients associated with exposure to fine particulate matter: A Korean cohort study

**DOI:** 10.1111/1759-7714.15070

**Published:** 2023-08-11

**Authors:** In‐Jae Oh, Cheol‐Kyu Park, Kyoung‐Bok Min, Jin‐Young Min, Chaeuk Chung, Seong‐Hoon Yoon, Changsoo Kim, Sei‐Hoon Yang

**Affiliations:** ^1^ Department of Internal Medicine Chonnam National University Medical School and Hwasun Hospital Hwasun South Korea; ^2^ Department of Preventive Medicine Colleague of Medicine, Seoul National University Seoul South Korea; ^3^ Institute of Health and Environment Seoul National University Seoul South Korea; ^4^ Department of Internal Medicine, College of Medicine Chungnam National University Daejeon South Korea; ^5^ Department of Internal Medicine Pusan National University Yangsan Hospital Yangsan South Korea; ^6^ Department of Preventive Medicine Yonsei University College of Medicine Seoul South Korea; ^7^ Department of Internal Medicine Wonkwang University School of Medicine Iksan South Korea

**Keywords:** healthcare utilization, lung cancer, particulate matter

## Abstract

**Background:**

Higher concentrations of particulate matter (PM) have been shown to cause deterioration of the symptoms of respiratory and cardiovascular disease in several regional studies. Here, we aimed to investigate the healthcare utilization of lung cancer patients associated with short‐term exposure to PM at the national level in Korea.

**Methods:**

We extracted the data of 210 558 subjects over a period of 3 years (2015–2017), who were diagnosed with lung cancer before 2015 and benefited from the National Health Insurance Sharing Service. We performed the interpolation method using the geographic information system to calculate the estimated mean PM_2.5_ and PM_10_ concentrations by regions and classified three groups as high (upper 10%), intermediate (10%–90%), and low (bottom 10%) based on the mean PM mass concentrations of the month.

**Results:**

The monthly average number of outpatient visits was significantly increased in high PM_2.5_ urban areas (46.296 vs. 50.646, *p* = 0.015). In high PM_2.5_ nationwide regions, the monthly average number of emergency admission was significantly increased (0.528 vs. 0.785, *p* = 0.001). The outpatient visits tended to change with PM_2.5_ concentration and correlated with PM_10_/PM_2.5_ concentrations in urban and nationwide areas. In high PM_2.5_ urban regions, there was a significant increase in bronchodilator prescriptions (3.102 vs. 3.758, *p* = 0.008). Concerning high PM_2.5_ nationwide regions, there were significantly increased prescriptions of antibiotics, steroids, bronchodilators, antihistamines, and mucolytics.

**Conclusions:**

This study suggests that exposure to PM_2.5_ is significantly associated with hospital utilization and drug prescription in lung cancer patients.

## Graphical abstract

We aimed to investigate the healthcare utilization of lung cancer patients associated with short‐term exposure to PM at the national level in Korea. The results suggest that exposure to PM_2.5_ is significantly associated with hospital utilization and drug prescription in lung cancer patients.

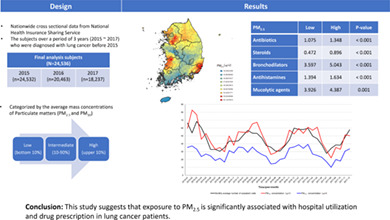

## INTRODUCTION

Particulate matter (PM) is suspended dust that has a diameter of <10 μm (PM_10_) and can be inhaled by humans and deposited in the lungs, particularly the alveoli. In addition to PM_10_, PM with diameter <2.5 μm (PM_2.5_) and <0.1 μm (PM_0.1_) is classified as fine and ultrafine PM, respectively.^1^ PM can cause various diseases including respiratory, allergic, cardiocerebrovascular, neurological, and psychiatric diseases. Furthermore, higher concentrations of PM cause deterioration of the symptoms of respiratory and cardiovascular disease as well as increase the associated risk, incidence, and mortality.^2^, ^3^ Short‐term exposure to PM_2.5_ also increases the risk for hospital admission for cardiovascular and respiratory diseases.^4^, ^5^, ^6^ Therefore, reduction of the total amount of PM is considered to be important for vulnerable patients, and proper early management of exacerbated patients will be needed in the future.^1^

Medical deterioration due to PM affects healthcare utilization. In a previous study, 10 μm/m^3^ elevation in PM_2.5_ concentration increased the risk of hospital admissions by 1.39% for citizens aged 65 years and older in 204 US urban counties during 1999–2002.^4^ In another study, it was shown that 10 μm/m^3^ elevation in two‐day averaged PM_2.5_ concentration increased the risk of hospital admissions due to respiratory disease by 2.07% in 26 US communities during 2000–2003.^5^ In addition, a study showed a 1.15% increase of respiratory admissions per 10 μm/m^3^ increase in PM_2.5_ mass in southern Europe.^6^ In Korea, for 10 μm/m^3^ increase in PM_2.5_ concentration, there was an 8.84% (95% confidence interval [CI]: 6.09–11.66%) increase in the risk of respiratory admissions for people aged 65 years and older in Seoul.^7^ Moreover, there was a 0.46% increase in respiratory outpatient visits and a 0.05% increase in respiratory admissions per 1% increase in PM_2.5_.^8^ A multivariate analysis showed a significant increase in respiratory‐related admissions with increasing PM levels and a decreasing relative humidity. Higher PM_2.5_ levels had a greater effect on respiratory‐related hospital admission than did PM_10_ levels. Children and the elderly were the most susceptible to hospital admission for respiratory disease.^9^ However, the previous studies have examined only specific provinces in Korea and the general population.

Thus, in this study, we aimed to investigate the healthcare utilization of lung cancer patients associated with short‐term exposure to PM at the national level in Korea. Specifically, we investigated the association between hospital utilization and PM by using 3 years of the National Health Insurance Service (NHIS) database of patients who were diagnosed with lung cancer.

## METHODS

### Patients and study design

NHIS provides mandatory national health insurance services to all citizens through government subsidies in Korea. Based on this service, they provide National Health Insurance Sharing Service (NHISS) and support research related to national health data.

**FIGURE 1:**
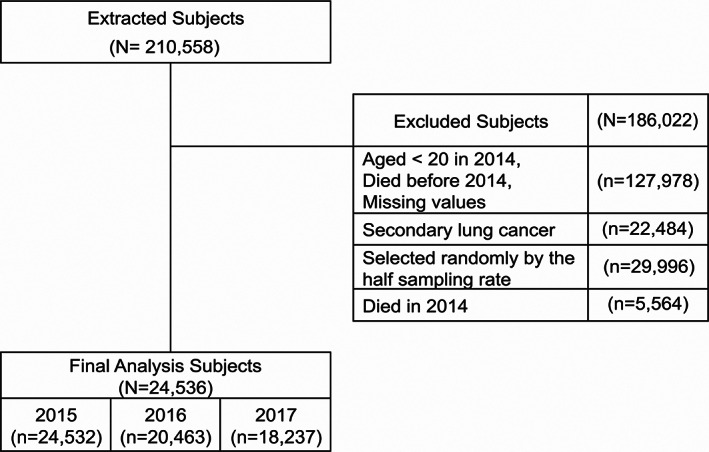
Study population.

We extracted the data of 210 558 subjects over a period of 3 years (2015–2017), who were diagnosed with lung cancer before January 1, 2015 and benefited from NHISS (Figure 1). We excluded 127 978 subjects who were aged <20 in 2014 or died before 2014 or missing values; 22 484 subjects with secondary lung malignancy; 29 996 subjects who were randomly half sampled; and 5564 subjects who died in 2014. Finally, 24 536 remaining subjects were eligible for our analysis. In each year, the number of subjects varied due to a lack of follow‐up during the period.

The trial was conducted in accordance with the Declaration of Helsinki (as revised in 2013). The data in the present study were extracted from the research database provided by NHISS and approved by the NHIS review board (NHIS‐2018‐1‐215).

### Variables

The PM mass concentration data were obtained from the National Institute of Environmental Research (NIER), which provides air pollution measurement data. We used annual mean PM_2.5_ and PM_10_ as measurements of exposure to fine PM. Frequencies of respiratory care admission, outpatient visit, emergency admission, and prescription were chosen as the outcome variables. The respiratory diseases were chosen according to seventh edition of Korean standard classification of disease (KCD‐7) including acute upper respiratory infections; pneumonia; other acute lower respiratory infections; other diseases of upper respiratory tract; chronic lower respiratory diseases; pulmonary diseases due to external agents; other respiratory diseases principally affecting the interstitium; suppurative and necrotic conditions of the lower respiratory tract; other diseases of pleura; and other diseases of the respiratory system. Demographic information included sex (male or female), age (20s, 30s, 40s, 50s, 60s, 70s and 80s), and regions (17 regions defined as metropolis or province, Table S1).

### Statistical analysis

We performed the interpolation method using the geographic information system (GIS) to calculate the estimated mean PM_2.5_ and PM_10_ concentrations by regions. PM_2.5_ and PM_10_ concentrations were the highest in February during 2015, the first year of the study period. Therefore, we classified three groups as high (upper 10%), intermediate (10%–90%), and low (bottom 10%) based on the mean PM mass concentrations of the month. We used the Scheffe test for the two extremes (high and low levels) to examine the effects of exposure to PM on hospital utilization. The statistical analyses were performed using SAS (version 9.4; SAS Institute), and a *p*‐value <0.05 was considered statistically significant.

## RESULTS

### Average mass concentrations of PM in 3 years

**FIGURE 2:**
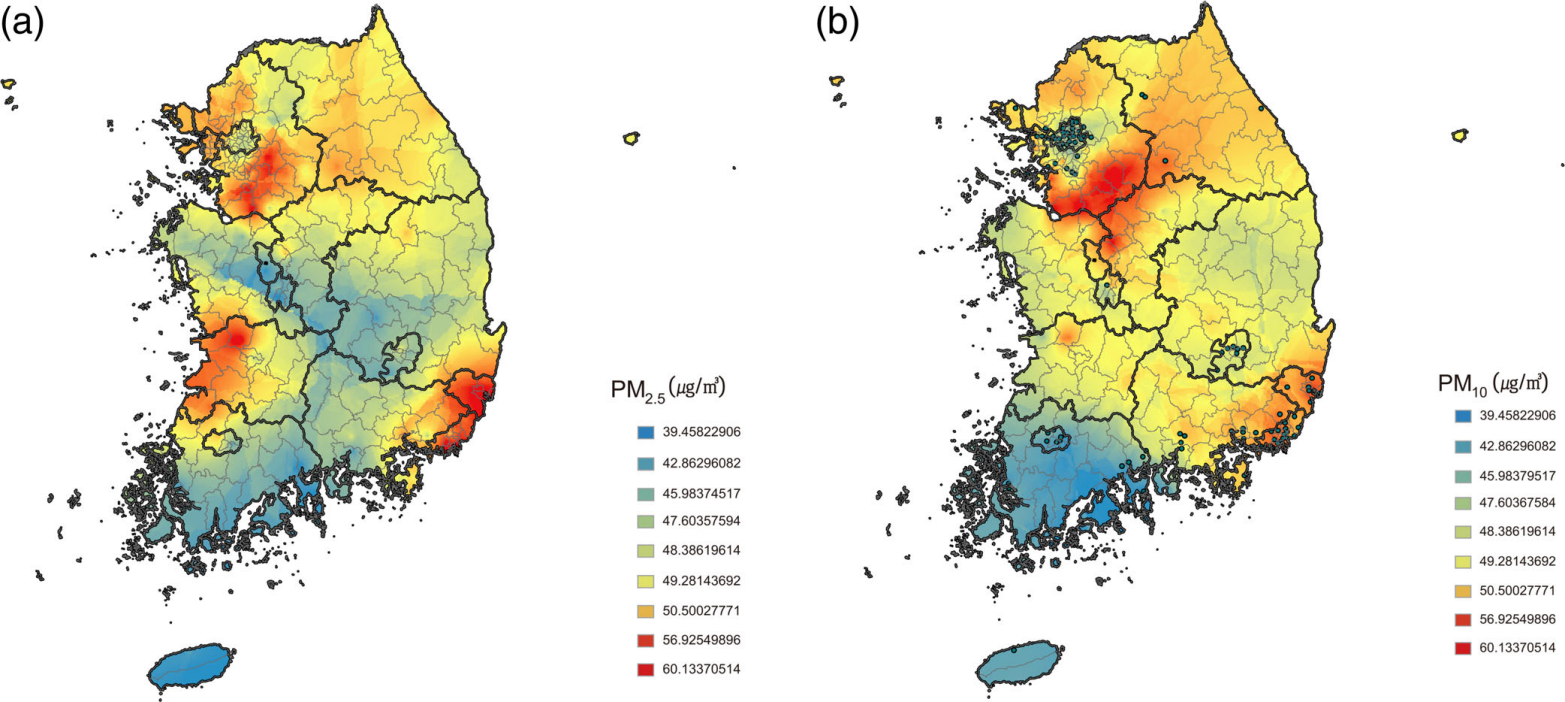
National contour graph of average mass concentrations of (a) PM_2.5_ and (b) PM_10_ in 3 years. Spatial and temporal distribution of the concentrations were calculated by spatial interpolation methods using geographic information system.

We introduced the GIS analysis method for PM mass concentrations data. Figure 2 is the contour graph of average PM mass concentrations for the years 2015–2017. Gyeonggi‐do, Chungcheongnam‐do, Jeollabuk‐do, Ulsan, and Busan had relatively higher PM_2.5_ concentrations, and Gyeonggi‐do, Chungcheongbuk‐do, Ulsan, and Busan had higher PM_10_ concentrations. The lowest concentration of PM_2.5_ and PM_10_ was observed in Jeollanam‐do.

### Associations between mass concentrations of PM and frequency of hospital utilization

We categorized the regions as urban (74 regions) and nationwide (253 regions), and the average mass concentrations of PM_2.5_ and PM_10_ in February 2015 as high (upper 10%), intermediate (10%–90%), and low (bottom 10%) group. The values in admission and outpatient visit were defined as monthly average counts per hundred patients. We conducted Scheffe test including high and low PM concentrations (Table 1).

**TABLE 1:** Average frequency of hospital utilization^a^ by mass concentrations of PM_2.5_ and PM_10_ ^b^ in 3 years.

Urban				Nationwide			
PM_10_	Low	High	*p*‐value^c^	PM_10_	Low	High	*p*‐value
Emergency admission	0.458	0.585	0.324	Emergency admission	0.695	0.788	0.407
Inpatient admission	1.554	1.150	<0.009	Inpatient admission	2.787	1.190	<0.001
Outpatient visit	47.179	47.883	0.895	Outpatient visit	58.85	43.114	<0.001
Total	49.19	49.618	0.962	Total	62.333	45.091	<0.001

Urban				Nationwide			
PM_2.5_	Low	High	*p*‐value	PM_2.5_	Low	High	*p*‐value
Emergency admission	0.466	0.475	0.995	Emergency admission	0.528	0.785	0.001
Inpatient admission	1.136	1.113	0.986	Inpatient admission	1.342	1.529	0.234
Outpatient visit	46.296	50.646	0.015	Outpatient visit	51.178	45.923	<0.001
Total	47.897	52.234	0.019	Total	53.048	48.236	<0.001

^a^ Monthly average number of admissions or visits per hundred patients.

^b^ We categorized mass concentrations of PM_2.5_ and PM_10_ in February 2015 into three groups: high (upper 10%), intermediate (10%–90%), and low (bottom 10%). In this table, the post hoc analysis between the high and low concentration groups is presented.

^c^ *p*‐value was calculated by Scheffe test.

Regarding PM_10_ and urban regions, the monthly average number of inpatient admissions was significantly different between two groups (low: 1.554 vs. high: 1.150, *p* = 0.009). In connection with the nationwide regions and PM_10_, monthly average numbers of inpatient admissions (2.787 vs. 1.19, *p* < 0.001) and outpatient visits (58.85 vs. 43.114, *p* < 0.001) were significantly different. In total, we found that as the level of PM_10_ concentration increased nationwide, the monthly average number of hospital utilization decreased (62.333 vs. 45.091, *p* < 0.001).

Concerning urban regions and PM_2.5_, the monthly average number of outpatient visits was significantly increased in high group (46.296 vs. 50.646, *p* = 0.015). Concerning PM_2.5_ and nationwide regions, monthly average numbers of emergency admissions (0.528 vs. 0.785, *p* = 0.001) and outpatient visits (51.178 vs. 45.923, *p* < 0.001) were significantly different between the two groups.

### Associations between mass concentrations of PM and frequency of drug prescription

In the urban regions, we found that the average monthly numbers of prescriptions differed significantly between high and low PM_10_ concentrations for three categories of drugs: steroids (0.353 vs. 0.638, *p* < 0.001), antihistamines (1.147 vs. 1.373, *p* = 0.014), and mucolytic agents (3.331 vs. 4.023, *p* < 0.001) (Table 2). Concerning nationwide regions, we noticed a statistically significant difference between low and high PM_10_ levels in antibiotics (1.283 vs. 1.148, *p* = 0.019), bronchodilators (5.277 vs. 4.222, *p* < 0.001), antihistamines (1.387 vs. 1.591, *p* = 0.002), and mucolytic agents (4.661 vs. 4.239, *p* = 0.004).

**TABLE 2:** Average frequency of drug prescription^a^ in 3 years by mass concentrations of PM_2.5_ and PM_10_ ^b^.

Urban				Nationwide			
PM_10_	Low	High	*p*‐value^c^	PM_10_	Low	High	*p*‐value
Antibiotics	0.989	1.130	0.080	Antibiotics	1.283	1.148	0.019
Steroids	0.353	0.638	<0.001	Steroids	0.611	0.700	0.113
Bronchodilators	3.300	3.168	0.824	Bronchodilators	5.277	4.222	<0.001
Antihistamines	1.147	1.373	0.014	Antihistamines	1.387	1.591	0.002
Mucolytic agents	3.331	4.023	<0.001	Mucolytic agents	4.661	4.239	0.004

Urban				Nationwide			
PM_2.5_	Low	High	*p*‐value	PM_2.5_	Low	High	*p*‐value
Antibiotics	1.067	1.007	0.636	Antibiotics	1.075	1.348	<0.001
Steroids	0.510	0.418	0.108	Steroids	0.472	0.896	<0.001
Bronchodilators	3.102	3.758	0.008	Bronchodilators	3.597	5.043	<0.001
Antihistamines	1.362	1.465	0.416	Antihistamines	1.394	1.634	<0.001
Mucolytic agents	3.359	3.777	0.059	Mucolytic agents	3.926	4.387	0.001

^a^ Monthly average number of drug prescriptions per patient.

^b^ We categorized mass concentrations of PM_2.5_ and PM_10_ in February 2015 into three groups: high (upper 10%), intermediate (10%–90%), and low (bottom 10%). In this table, the post hoc analysis between the high and low concentration groups is presented.

^c^ *p*‐value was calculated by Scheffe test.

Regarding PM_2.5_ and urban regions, there was a significant increase in bronchodilator prescriptions (3.102 vs. 3.758, *p* = 0.008). The mean count of mucolytic agents showed increasing tendency between high and low groups (3.359 vs. 3.777, *p* = 0.059). Concerning the nationwide regions and PM_2.5_, the mean counts of all drugs showed statistically significant increase in high PM_2.5_ group; antibiotics (1.075 vs. 1.348, *p* < 0.001), steroids (0.472 vs. 0.896, *p* < 0.001), bronchodilators (3.597 vs. 5.043, *p* < 0.001), antihistamines (1.394 vs. 1.634, *p* < 0.001), and mucolytic agents (3.926 vs. 4.387, *p* = 0.001).

### Trend of outpatient visits in high PM concentration group by the levels of PM_2_ _.5_ / PM_10_

**FIGURE 3:**
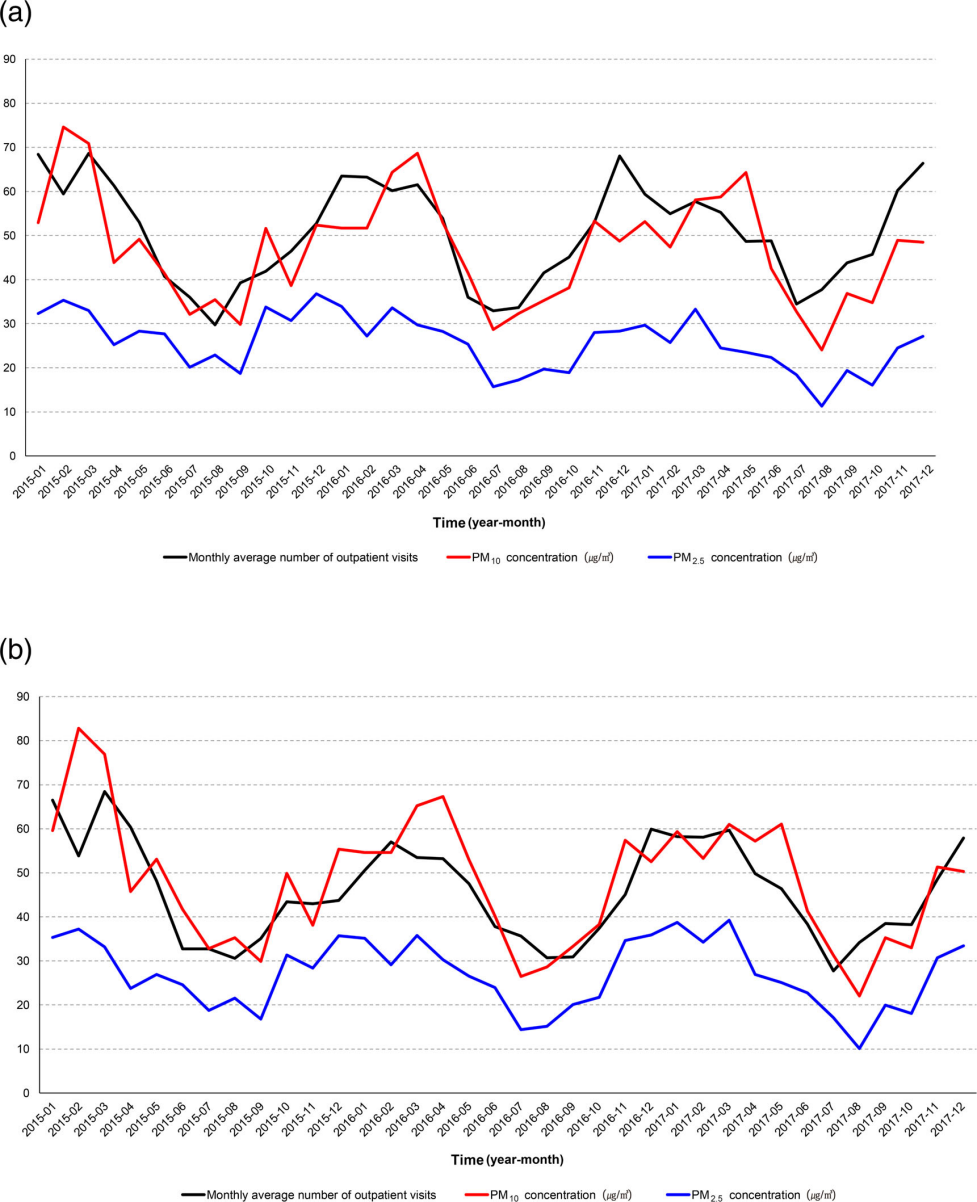
Trends in outpatient visits with levels of PM_2.5_ and PM_10_ in (a) urban areas and (b) nationwide.

As we found that the number of outpatient visits was significantly associated with PM_2.5_ in the previous analysis, we plotted the monthly average number of outpatient visits based on the regions (urban/nationwide) and PM_2.5_ and PM_10_ concentrations in the high PM concentration groups (based on the levels of PM_2.5_ in February 2015) in 3 years (Figure 3). The plots suggest that the monthly average numbers of outpatient visits move up and down along with PM_2.5_ concentration regardless of its level.

### Correlation between mass concentrations of PM and the number of outpatient visits

**FIGURE 4:**
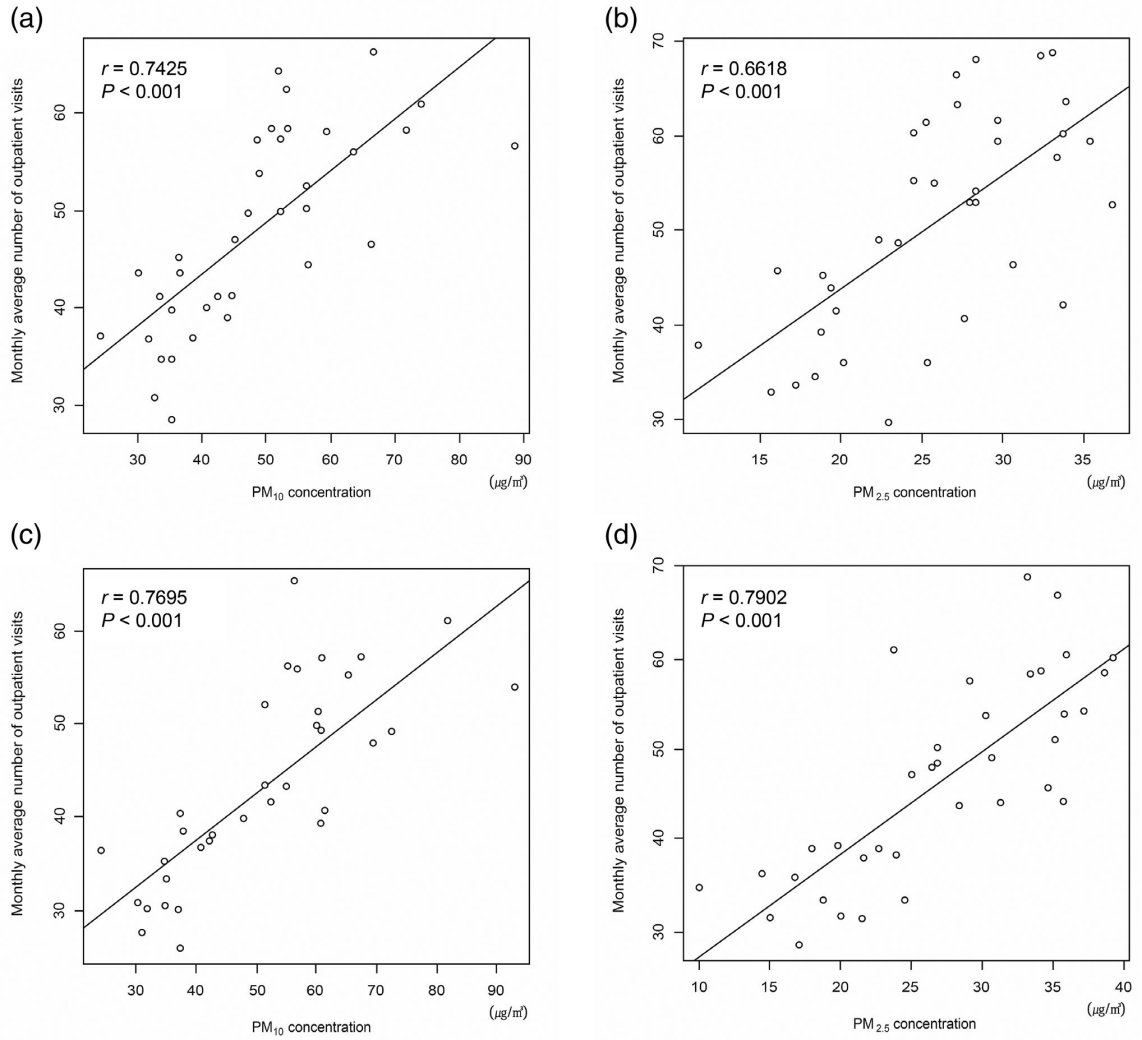
Correlation graph between particulate matter (PM) concentrations and the number of outpatient visits in (a) urban high PM_10_, (b) urban high PM_2.5_, (c) nationwide high PM_10_, and (d) nationwide high PM_2.5_ areas.

Figure 4 shows the correlation between monthly average numbers of outpatient visits related to respiratory disease and PM_2.5_ and PM_10_ level in the high PM concentration groups by the levels of PM_2.5_ in February 2015. In urban areas, the number of outpatient visits showed positive correlations with the level of PM_10_ (*r* = 0.7425, *p* < 0.001) and PM_2.5_ concentration (*r* = 0.6618, *p* < 0.001). With regard to nationwide regions, the positive correlations were also observed between the number of appointments and the levels of PM_10_ and PM_2.5_ concentration (*p* < 0.001 in both).

## DISCUSSION

In this cohort study in Korea, we stratified by PM concentration and region to examine associations between levels of PM concentrations and hospital utilization during a period of 3 years. Overall, we found that higher levels of PM_2.5_ and PM_10_ are associated with higher rates of all types of hospital utilization and prescriptions due to an increase of respiratory disease. Although the health care utilization according to PM_10_ concentration was difficult to interpret because of the various factors that we could not include in this analysis, PM_2.5_'s harmful effects on lung cancer patients' health seem more obvious. In urban regions, we found that as the level of PM_2.5_ concentration increased, the number of outpatient visits also increased (*p* = 0.015). Although the number of admissions (emergency or inpatient) did not differ according to high versus low PM_2.5_ concentration, the overall hospital utilization showed statistical significance (*p* = 0.019) because of the outpatient visits. However, regarding the nationwide regions, the results were reversed. As the level of PM_2.5_ concentration increased, the number of outpatient visits decreased (*p* < 0.001). One reason for this reversal relates to the number of outpatient visits in the other regions except urban regions.

We reasonably assumed that healthcare utilization is related to the number of prescriptions; hence, we also investigated the associations between PM mass and the number of prescriptions. Regarding urban regions, the level of PM_10_ was significantly associated with a higher rate of steroids, antihistamines, and mucolytic agents. However, the high PM_2.5_ concentration was significantly associated with an increased prescription of antibiotics, steroids, bronchodilators, antihistamines, and mucolytics nationwide. In particular, the prescription of bronchodilators significantly increased only in the high PM_2.5_ concentration group, unlike the PM_10_ concentration group in urban regions and nationwide. This phenomenon might be because only PM_2.5_ can reach alveoli and bronchioles.

In exploratory data analysis, we performed correlation analysis and visualized trends between PM mass and the number of outpatient visits. Across all levels of PM, we found a strong positive correlation (*p* < 0.001). Furthermore, the trends in outpatient visits also moved along with the value of PM. Linear regression also showed that hospital utilization increased significantly according to increase of the concentration of PM_2.5_ and PM_10_ in urban regions and nationwide.

Exposure to PM leads to increased pulmonary inflammation and respiratory symptoms aggravated by oxidative stress and direct toxic injury.^1^, ^10^, ^11^, ^12^ This is particularly dangerous for patients with pre‐existing respiratory diseases, as exposure to PM can lead to acute exacerbation of their ailment. It has also been reported that long‐term exposure to high concentrations of PM increases the prevalence of chronic obstructive pulmonary disease and lung cancer in adults, leading to a decline in pulmonary function.^1^, ^12^ The high concentration of PM in the atmosphere has a profound effect on the prevalence of chronic respiratory diseases and the risk of acute exacerbation.^13^ Additionally, PM_2.5_ exposure is associated with a long recovery time, more leading to an increase in both the mortality rate and the overall medical burden.^1^ Therefore, it is considerably important that effective policies and medical practices are put into place to minimize the public health risks associated with PM exposure.

In 2005, as outlined by the World Health Organization (WHO), the maximum acceptable annual average concentration of PM_10_ was ≤20 μg/m^3^, with a limit of ≤50 μg/m^3^ per 24‐h period. In Korea, the annual and daily average concentrations of PM_10_ are <50 and < 100 μg/m^3^, respectively. The annual and daily average concentrations of PM_2.5_ are <15 and 35 μg/m^3^, respectively. Overall concentrations of PM_10_ and PM_2.5_ are above 81 μg/m^3^ and 36 μg/m^3^, respectively, and therefore the PM concentration forecast grade is designated as “bad” in Korea.^14^

In 2013, the International Agency for Research on Cancer (IARC), an intergovernmental agency forming part of WHO, classified outdoor air pollution as a Group 1 agent—carcinogenic to humans. In Europe, a multinational prospective cohort study showed a hazard ratio (HR) of 1.22 per 10 μm/m^3^ increase in PM_10_, and an HR of 1.18 per 5 μm/m^3^ increase in PM_2.5_ for lung cancer.^15^ In a comprehensive meta‐analysis conducted in Korea in 2015, the risk of lung cancer increased by 1.09‐fold (95% CI: 1.01–1.14) when the concentration of PM_2.5_ increased by 10 μg/m^3^. A correlation between PM_10_ concentration and lung cancer incidence was also observed; however, the correlation was comparatively weak compared to that between lung cancer risk and PM_2.5_ concentration (1.08‐fold increased risk; 95% CI: 1.00–1.17).^14^ In addition, lung cancer incidence was higher in smokers who were exposed to high amounts of PM_2.5_; it was confirmed that fine PM affects smokers' lung cancer development to a significantly greater degree relative to healthy people.^10^, ^16^ It is estimated that about 500 000 lung cancer deaths can be attributed to air pollution.^17^ Both PM_10_ and PM_2.5_ were reported to significantly increase the mortality rate of lung cancer patients in 2017 and 2018 meta‐analyses.^18^ Thus, to reduce lung cancer prevalence and mortality, control of PM generation and avoidance of PM exposure, together with smoking cessation, are of the utmost importance.^1^, ^19^

This study had several limitations. First, although this was a nationwide large‐scale study, regional and environmental factors should be considered when applied outside of Korea. Second, unlike with PM_2.5_, consistent results were not shown for PM_10_ concentration and healthcare utilization rate, which requires consideration for a wider range of environmental factors. Third, the association between PM concentration and the frequency of drug prescription for respiratory symptom control was possible, but we could not present any relevance to ongoing lung cancer treatment (e.g., chemotherapy or radiation therapy). Fourth, we did not perform the subanalysis of a specific group such as gender in which PM may have more impact. Female patients tend to have smaller absolute lung volumes, and the proportions of smokers and pathological subtypes are different when compared to their male counterparts.

In conclusion, this study suggests that exposure to PM_2.5_ is significantly associated with hospital utilization and drug prescription in lung cancer patients although we did not obtain long‐term PM data. Moreover, we used large data based on NHISS, so that our study can be used as a reference for the national healthcare policy.

## AUTHOR CONTRIBUTIONS

Conceptualization: Oh IJ, Yang SH. Data curation: Oh IJ, Yang SH. Formal analysis: Oh IJ, Yang SH Funding acquisition: Oh IJ. Investigation: Oh IJ, Park CK, Chung C, Yoon SH, Yang SH. Methodology: Oh IJ, Kim CS, Yang SH. Software: Oh IJ. Validation: Kim CS. Visualization: Min KB, Min JY. Writing—original draft: Oh IJ. Writing—review and editing: Oh IJ, Park CK, Chung C, Yoon SH, Yang SH.

## FUNDING INFORMATION

This work was supported by Daewon Pharmaceutical Co., Ltd., Seoul, Republic of Korea. The funder had no role in the study design, data collection and analysis, decision to publish, or preparation of the manuscript.

## CONFLICT OF INTEREST STATEMENT

The authors declare that they have no conflicts of interest.

## Supporting information


**TABLE S1.** Descriptive characteristics of study population (2015–2017).
