# Supplementary material for: Healthcare utilization of lung cancer patients associated with exposure to fine particulate matter: A Korean cohort study
Source: Thorac Cancer. 2023 Aug 11;14(27):2777–84. doi: 10.1111/1759-7714.15070 (PMC10518228; doi:10.1111/1759-7714.15070)
Supplement: Supplementary file 1 — TABLE S1. Descriptive characteristics of study population (2015–2017). [file TCA-14-2777-s001.docx]

**Supplementary Table 1.** Descriptive characteristics of study population (2015 – 2017)

|  | 2015 - 2017 | |
| --- | --- | --- |
|  | N | % |
|  | 24,536 | 100 |
| **Sex** |  |  |
| Male | 15,748 | 64.18 |
| Female | 8,788 | 35.82 |
| **Age** |  |  |
| 20s | 55 | 0.22 |
| 30s | 212 | 0.86 |
| 40s | 1,005 | 4.1 |
| 50s | 3,889 | 15.85 |
| 60s | 7,555 | 30.79 |
| 70s | 8,594 | 35.03 |
| 80s | 3,226 | 13.15 |
| **Regions (17 Metropolis and Provinces)** |  |  |
| Seoul | 4,830 | 19.69 |
| Busan | 1,434 | 5.84 |
| Incheon | 1,124 | 4.58 |
| Daegu | 1,203 | 4.9 |
| Gwangju | 665 | 2.71 |
| Daejeon | 595 | 2.43 |
| Ulsan | 427 | 1.74 |
| Sejong | 77 | 0.31 |
| Gyeonggi-do | 5,380 | 21.93 |
| Gangwon-do | 850 | 3.46 |
| Gyeongsangbuk-do | 1,690 | 6.89 |
| Gyeongsangnam-do | 1,468 | 5.98 |
| Chungcheongbuk-do | 755 | 3.08 |
| Chungcheongnam-do | 1,190 | 4.85 |
| Jeollabuk-do | 1,113 | 4.54 |
| Jeollanam-do | 1,434 | 5.84 |
| Jeju | 301 | 1.23 |
